# Associations Between the Brain Care Score and Structural Brain Changes and Cognitive Impairment Using Data From the Hamburg City Health Study

**DOI:** 10.1111/ene.70796

**Published:** 2026-10-22

**Authors:** Jenna Schellin, Susanne Sehner, Marie Neumann, Marvin Petersen, Felix Leonard Nägele, Sanjula D. Singh, Jonathan Rosand, Nils Schumacher, Birgit‐Christiane Zyriax, Raphael Twerenbold, Bastian Cheng, Götz Thomalla, Märit Jensen

**Affiliations:** ^1^ Department of Neurology University Medical Center Hamburg‐Eppendorf (UKE) Hamburg Germany; ^2^ Department of Cardiology University Medical Center Hamburg‐Eppendorf (UKE) Hamburg Germany; ^3^ Brain Care Labs, Department of Neurology, Harvard Medical School Massachusetts General Hospital Boston Massachusetts USA; ^4^ Broad Institute of MIT and Harvard Cambridge Massachusetts USA; ^5^ Institute of Human Movement Science, Science and Transfer Center Active City University of Hamburg Hamburg Germany; ^6^ Research Group Preventive Medicine and Nutrition, Midwifery Science, Health Care Research and Prevention University Medical Center Hamburg‐Eppendorf (UKE) Hamburg Germany

**Keywords:** Brain Care Score, brain health, cognition, neuroimaging, structural brain changes

## Abstract

**Background:**

The Brain Care Score (BCS) was designed to engage patients in behavior change targeting modifiable risk factors for dementia, stroke, and depression. We investigated its associations with structural brain changes and cognitive function to evaluate its utility for early prevention.

**Methods:**

We analyzed data from the population‐based Hamburg City Health Study (participants aged 45–74 years). A modified BCS (range 0–18) was derived from physiological, lifestyle, and social factors. Brain MRI including diffusion tensor imaging provided markers of neurodegeneration, vascular brain damage, and structural brain network organization. Cognitive function was assessed using the MMSE and CERAD‐Plus subtests.

**Results:**

Among 2453 participants (mean age 63.8 years, 44% women, median BCS 11), a higher BCS was associated with less vascular brain damage: a 5‐point‐increase was associated with lower white matter hyperintensity burden (relative increase 0.81; 95% CI [0.75, 0.87]) and lower Peak Width of Skeletonized Mean Diffusivity (relative increase 0.97; 95% CI [0.96, 0.98]). A higher BCS was also associated with better structural connectivity (higher global efficiency, lower clustering coefficient). Regarding cognition, a 5‐point‐increase was associated with faster processing speed (relative increase 0.96, 95% CI [0.94, 0.98]), better executive function (relative increase 0.94, 95% CI [0.91, 0.97]), and improved verbal fluency (absolute increase 0.57, 95% CI [0.05, 1.10]).

**Conclusion:**

The BCS represents a simple score capturing modifiable risk factors for common age‐related brain disease, adaptable to large population‐based cohorts. Higher scores are associated with healthier brain structure, network organization, and better cognitive function, supporting its role in guiding preventive interventions.

**Trial Registration:**

ClinicalTrials.gov number, NCT03934957

## Graphical abstract

In this analysis of 2453 participants from the Hamburg City Health Study, a higher Brain Care Score (BCS) was associated with less vascular brain damage (lower white matter hyperintensity burden, lower Peak Width of Skeletonized Mean Diffusivity). A higher BCS was further associated with preserved brain network organization and better performance across multiple cognitive domains. These findings suggest that the BCS may serve as a practical tool for preventive strategies against cognitive decline by capturing modifiable determinants of brain disease.

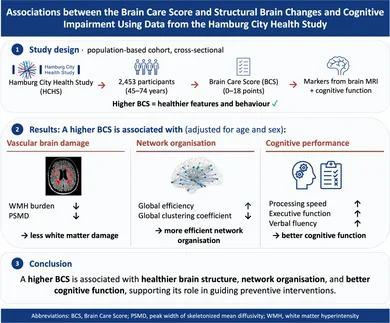

## 1 Introduction

Dementia is a major public health challenge, with the prevalence of all‐cause dementia estimated at over 57 million people globally in 2021, and numbers projected to approximately triple by 2050 due to population aging [1]. It exerts profound personal and societal costs, underscoring the urgent need for effective preventive strategies [2].

Structural brain changes are key mediators in the development of cognitive decline, with both vascular and neurodegenerative factors contributing to an increased risk of stroke and dementia [3]. Vascular risk factors, such as hypertension and diabetes, can lead to cerebral small vessel disease (CSVD), which manifests as microvascular lesions, including white matter hyperintensities (WMH), lacunes, and microbleeds that disrupt brain connectivity and compromise the brain's structural integrity [4]. At the same time, neurodegenerative processes cause neuronal loss, synaptic dysfunction, and cortical thinning, thereby also impairing cognitive function [5]. The brain is organized in structural and functional networks of interconnected brain regions, and CSVD comes along with alterations of brain connectivity and brain network properties [6, 7]. Imaging markers of CSVD often precede cognitive decline [8], and their progression is strongly influenced by modifiable risk factors. Importantly, up to 40% of dementia cases are attributable to preventable exposures [9, 10, 11]. However, effective tools to translate this knowledge into personalized preventive strategies remain limited.

To address this gap, the Brain Care Score (BCS) was developed as a pragmatic, evidence‐based composite index encompassing physiological, lifestyle, and social factors [12]. Traditional vascular risk scores are typically restricted to well‐established cardiovascular risk factors [13, 14]. By capturing the effects of multiple domains associated with brain health, the BCS by design provides a broader perspective. In contrast to these established scores, the BCS not only incorporates conventional cardiovascular risk factors, such as hypertension and diabetes, but also emphasizes lifestyle factors such as nutrition, sleep, and stress. Moreover, it was designed as a “positive” score, where higher values reflect healthier features and behavior rather than increased risk in order to prioritize motivational aspects.

Prior analyses in the UK Biobank demonstrated that a higher BCS is associated with reduced risk of dementia and stroke, suggesting its potential utility for prevention [12, 15]. In the same population, individuals with higher BCS values exhibited lower WMH burden and slower progression of white matter abnormalities over time [16]. Findings from the Women's Health Study showed a lower risk of cerebrovascular events with a higher baseline BCS [17].

This study aims to validate and further investigate the association between the BCS and structural brain changes including imaging markers of neurodegeneration and vascular brain damage, as well as its relationship with cognitive performance in a population‐based middle‐aged to elderly cohort. It amends previous research by also including measures of brain network organization. Given the previously reported associations of the BCS with stroke and dementia risk [12], investigating its relationship with neuroimaging markers may provide further insight into the underlying mechanisms linking modifiable risk factors to cognitive decline.

## 2 Methods

### 2.1 Study Cohort

The Hamburg City Health Study (HCHS) is an ongoing large, prospective, population‐based cohort study with the goal of understanding major chronic diseases to enhance diagnostic and treatment options. A sample of 45,000 inhabitants of the city of Hamburg, Germany, aged 45–74 years are to be included. The participants are taking part in an extensive baseline assessment, including examinations targeting major organ system function and questionnaires regarding, among others, lifestyle, quality of life, medical history as well as healthcare utilization. Individuals were invited for an additional MRI examination of the brain, either randomly or based on increased cerebrovascular risk defined by a Framingham Stroke Risk Score > 7 [18]. From the first 10,000 participants, we included those who had available data to calculate the BCS and at least one measure of brain structure from brain MRI available.

### 2.2 Demographical Data and Medical History

All participants of the HCHS completed questionnaires on demographic information and medical history. Level of education was classified into three standardized levels according to the International Standard Classification of Education. In addition, all participants underwent an extensive baseline clinical examination, blood pressure evaluation, and blood sampling. Hypertension was defined as systolic/diastolic blood pressure ≥ 140/90 mmHg, use of antihypertensive medication, or self‐reported diagnosis of hypertension. Diabetes mellitus was defined as fasting glucose > 126 mg/dL, non‐fasting glucose > 200 mg/dL, or self‐reported diagnosis. Hypercholesterolemia was defined by an LDL/HDL ratio > 3.5 or lipid‐lowering medication. Metabolic syndrome was diagnosed according to the International Diabetes Federation consensus definition [19].

### 2.3 Calculation of the Brain Care Score (BCS)

The BCS is composed of three domains: physiological measures (blood pressure, hemoglobin A1c, cholesterol, and body mass index [BMI]), lifestyle factors (nutrition, alcohol consumption, smoking, aerobic activity and sleep), and social determinants (stress, social relationships, and meaning in life) [12]. In this study, the scoring system was adapted due to the absence of data on “social relationships” and “meaning in life,” resulting in the exclusion of these two items. The modified BCS in our cohort ranged from 0 to 18 points, with higher scores reflecting a more favorable brain health profile (Table 1).

**TABLE 1:** The Brain Care Score in the HCHS cohort.

Category	Criteria/description	Rank
Physical		
Blood pressure	Systolic or diastolic blood pressure of greater than 140/90 mmHg	0
	Systolic or diastolic blood pressure 120–140/80–90 mmHg	2
	Systolic and diastolic blood pressure lower than 120/80 mmHg	3
Blood glucose	Hemoglobin A1c greater than 6.4%	0
	Hemoglobin A1c between 5.7% and 6.4%	1
	Hemoglobin A1c less than 5.7%	2
Cholesterol	Total cholesterol 190 mg/dL or higher	0
	Total cholesterol less than 190 mg/dL	1
Body mass index	Between 18.5 and 25 kg/m^2^	2
	Higher than 25 and lower than 30 kg/m^2^	1
	Higher than or equal to 30 kg/m^2^	0
Lifestyle		
Nutrition	Dietary habits 4.5 or more servings of fruit and vegetables per day A red meat score of 1 or 2 3 or more servings of bread slices or cereal bowls per day Sometimes, rarely, or never add salt to a meal	
	Typical diet does not include at least 2 of the recommendations above	0
	Typical diet includes 2 of the recommendations above	1
	Typical diet includes 3 or more of the recommendations above	3
Alcohol consumption	Drinking ≥ 4 times/week	0
	Drinking 1–2 times/week or 1–3 times/month	1
	Drinking only on special occasions or never	2
Smoking	Current smoker	0
	Former or never smoker	2
Aerobic activities	< 150 min/week	0
	> 150 min/week	1
Sleep	Less than 7 h/day	0
	7 or more hours/day	1
Social emotional		
Stress	Self‐perceived tension, fidgetiness, or restlessness several days, more than half the days, or nearly every day in the last 2 week	0
	No self‐perceived tension, fidgetiness, or restlessness in the last 2 week	1

### 2.4 MRI Acquisition and Processing

All MRI data were obtained using a single 3T Siemens Skyra scanner (Siemens, Erlangen, Germany), applying an identical imaging protocol for all participants. Structural scans included a 3D T1‐weighted rapid acquisition gradient‐echo sequence (MPRAGE; 256 axial slices, echo time (TE) = 2.12 ms, slice thickness (ST) = 0.94 mm, repetition time (TR) = 2500 ms, in‐plane resolution (IPR) = 0.83 × 0.83 mm) and a 3D T2‐weighted fluid‐attenuated inversion recovery (FLAIR) image (192 axial slices, TR = 4700 ms, TE = 392 ms, 192 axial slices, ST = 0.9 mm, IPR = 0.75 × 0.75 mm). Diffusion‐weighted imaging (single‐shell diffusion MRI (dMRI)) comprised 75 axial slices covering the whole brain with diffusion gradients (b = 1000 s/mm^2^) in 64 noncollinear directions (TR = 8500 ms, TE = 75 ms, ST = 2 mm, IPR = 2 × 2 mm with anterior–posterior phase‐encoding).

Preprocessing followed previously described procedures with full documentation of the MRI processing pipeline available in a GitHub repository (https://github.com/csi‐hamburg/CSIframe/wiki) [20]. Briefly, dMRI and T1‐weighted images were processed with QSIPrep 0.14.2. This included intensity normalization, skull‐stripping, MP‐PCA denoising, removal of ringing artifacts, and correction for B1 field inhomogeneity, head motion, eddy current, and susceptibility‐related distortions [21].

Quantification of brain volume and cortical thickness was based on T1‐weighted images and performed using Freesurfer v.6.0.1 [22]. All resulting segmentations were visually and quantitatively inspected (with outliers defined as values > 2 standard deviations from the median), and data of insufficient quality (i.e., segmentation errors) was excluded.

WMH were segmented following previously described methods [20]. In short, the Brain Intensity AbNormality Classification Algorithm (BIANCA) algorithm with LOCally Adaptive Threshold Estimation (LOCATE) was applied to preprocessed FLAIR and T1‐weighted images [23]. The resulting masks were refined using Freesurfer output to remove non‐white matter structures (e.g., corpus callosum and basal ganglia). WMH lesion volumes were normalized by intracranial volume estimated via Freesurfer v.6.0.1 [22], the resulting measure is referred to as WMH load. Peak Width of Skeletonized Mean Diffusivity (PSMD) was computed following standard procedures, adapted by incorporating non‐linear registration to MNI space using Advanced Normalization Tools (ANTs) SyN registration [24, 25].

PSMD was defined as the difference between the 95th and 5th percentile of MD values across the white matter skeleton, with regions susceptible to partial volume effects of cerebrospinal fluid masked out.

Structural connectomes were reconstructed with QSIprep's “mrtrix_multishell_ss3t” workflow applied to preprocessed dMRI and T1‐weighted images (for details see [26]). Subsequent analyses were conducted using the brain connectivity (BCT) toolbox [27] in Python (bctpy). Processing included connectome normalization and calculation of global graph‐theoretical metrics. From each connectome global efficiency [28] and clustering coefficient [29] were extracted. The global efficiency quantifies the integrative capacity of the network as the average inverse shortest path length, that is, the length of a path between two nodes is the total weight of edges comprising that path, while the weighted clustering coefficient of a node reflects local interconnectivity based on the geometric mean of edge weights within node‐specific triangles. Averaging this metric across all nodes provides an index of the overall strength of local interconnectivity within the network and, consequently, its capacity for segregated information processing. Because both global efficiency and the clustering coefficient depend on low‐level features of the connectome, such as the distribution of connection weights and node degrees, their values can be influenced by basic network architecture rather than by meaningful topological organization. To mitigate this, both measures were normalized using graph parameters obtained from and averaged over 100 null models, generated by randomly rewiring each subject's connectome [30].

### 2.5 Assessment of Cognitive Function

Cognitive testing was administered by a trained study nurse and included the Mini‐Mental‐State Examination (MMSE) as well as the following subtests of the Consortium to Establish a Registry for Alzheimer's Disease Neuropsychological Assessment Battery (CERAD‐Plus) capturing different cognitive domains: the Animal Naming Test (verbal fluency), Trail Making Test A (processing speed) and B (executive function), and Word List Recall (verbal memory) [31].

### 2.6 Statistical Analysis

Descriptive statistics were used to characterize the study population, with continuous variables presented as mean with standard deviation or median with interquartile range based on their distribution, and categorical variables as absolute frequencies and percentages. To ensure the validity of statistical inference within the chosen parametric modeling framework, continuous variables with skewed distributions were log‐transformed prior to analysis.

In order to avoid a potential selection bias arising from missing responses to individual BCS items, the BCS was calculated using a hierarchical approach to missing data. At the subscale level, missing individual items, up to a maximum of four of five subscale items in a small subset of participants (Tables S4 and S5), were treated as indicating the absence of the respective protective factor (scored as zero). At the total scale level, a complete‐case approach was applied; the total BCS was only calculated for participants with valid data across all three subscales. For multi‐item subscales, in case of missing items, the subscale was calculated from the remaining items. The “stress” subscale is derived from a single item, so a missing response precludes calculation of this subscale entirely. Available‐case and complete‐case handling are therefore equivalent for this subscale. Of a total of 2618 participants with available MRI, 165 participants lacked a valid “stress” subscale value and were excluded from BCS calculation. One of these 165 participants also had a fully missing lifestyle subscale. For the other 2453 participants all subscales could be calculated (Figure 1, Tables S4–S6). To assess the robustness of this scoring approach, a sensitivity analysis was performed using a strict complete‐case population for all BCS items (Tables S1–S3).

**FIGURE 1:**
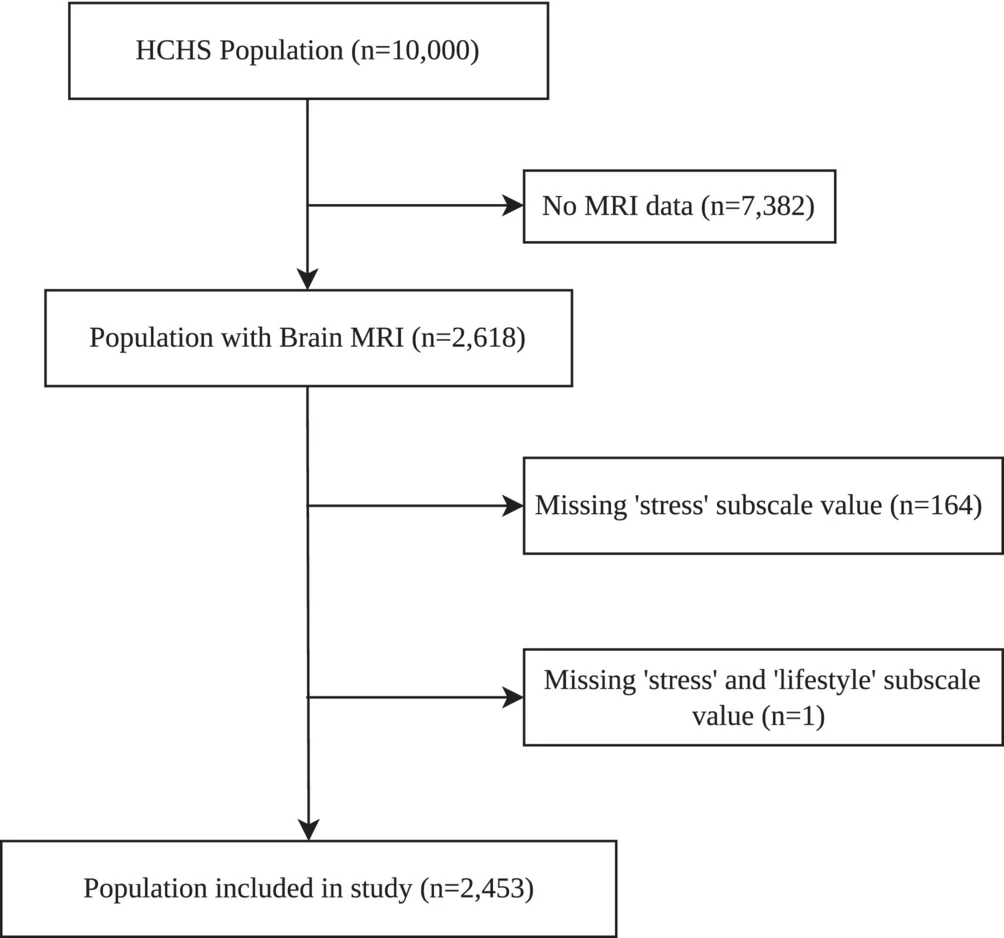
Flowchart of the study population.

The endpoints were neuroimaging measures (WMH volume, PSMD, total brain volume, and cortical thickness), measures of structural brain network organization (global efficiency, global clustering coefficient), and the different domains of cognitive function. Adjusted linear regression models were employed to evaluate the association between BCS and each clinical endpoint, accounting for age and sex as potential confounders and including their interaction term. To facilitate clinical interpretation, the independent variable BCS was scaled so that results represent the effect per 5‐unit increase. For untransformed outcomes, the absolute increase in the respective unit is reported, whereas for log‐transformed outcomes, results were back‐transformed and are reported as relative increases. The assumption of linearity was visually evaluated using local polynomial regression (local mean smoothing) with an Epanechnikov kernel function and a fixed bandwidth of 0.8 (Figures S1 and S2).

Given the exploratory nature of the study aiming at generating hypotheses rather than confirming pre‐specified effects, no correction for multiple testing was applied, and nominal *p*‐values are reported, with *p* < 0.05 considered statistically significant. Statistical analyses were performed using Stata Statistical Software, version 19.5 (StataCorp, College Station, TX, USA).

### 2.7 Standard Protocol Approvals, Registrations, and Patient Consents

The HCHS was approved by the Ethics Committee of the State of Hamburg Chamber of Medical Practitioners (Ethik‐Kommission Landesärztekammer Hamburg, PV5131) and has been registered on ClinicalTrials.gov (NCT03934957). All participants gave written informed consent.

## 3 Results

### 3.1 Study Population

Among the cohort of the first 10,000 participants enrolled in the HCHS, 2453 individuals who underwent brain MRI with at least one measure of structural brain changes and had available data to calculate the BCS were included in the final analysis (Figure 1). The mean age was 63.8 years (SD 8.4), and 1075 (44%) were women. Cardiovascular risk factors were common, with 72% of participants having hypertension, 27% having hypercholesterolemia, and 9% having diabetes. Participants had a median BCS of 11 (interquartile range [IQR] [9, 12]), indicating moderate adherence to brain‐healthy lifestyle factors. Detailed characteristics of the study population including neuroimaging findings are presented in Table 2.

**TABLE 2:** Description of study population (*N* = 2453).

Characteristic	
Age [years], mean (SD)	63.8 (8.4)
Female, *n*/*N* (%)	1075/2453 (43.8%)
Level of education, *n*/*N* (%)	
Low education	96/2369 (4.1%)
Medium education	1158/2369 (48.9%)
High education	1115/2369 (47.1%)
Medical history and risk factors, *n*/*N* (%)	
Arterial hypertension	1711/2372 (72.1%)
Diabetes mellitus	210/2296 (9.1%)
Hypercholesterolaemia	619/2311 (26.8%)
Atrial fibrillation	136/2250 (6.0%)
Metabolic syndrome	958/2174 (44.1%)
Smoking	
Never/past	2024/2446 (82.7%)
Current	422/2446 (17.3%)
History of myocardial infarction	85/2443 (3.5%)
History of heart failure	110/2268 (4.9%)
History of stroke	75/2439 (3.1%)
Brain Care Score (0–18), median (IQR)	11.0 (9.0, 12.0)
Category physical (potential range 0–8)	4.0 (3.0, 5.0)
Category lifestyle (potential range 0–9)	6.0 (4.0, 7.0)
Category social/emotional (potential range 0–1), *n* (%)	1 Point: 1963/2453 (80.0%) 0 Points: 490/2453 (20.0%)
Brain imaging parameters, median (IQR)	
Brain volume, normalized	0.80 (0.77, 0.83) [*n* = 2356]
WMH load [proportion of total brain volume ×10^3^]	0.99 (0.51, 1.98) [*n* = 2318]
PSMD [×10^3^]	0.22 (0.20, 0.25) [*n* = 2256]
Cortical thickness [mm]	2.61 (2.55, 2.67) [*n* = 2356]
Global efficiency	0.59 (0.57, 0.61) [*n* = 2144]
Global clustering coefficient	1.24 (1.20, 1.31) [*n* = 2144]
Cognitive assessment, median (IQR)	
Animal naming test (Verbal fluency) [*n* words]	24.0 (20.0, 29.0) [*n* = 2312]
Word list recall (Verbal memory) [*n* words]	8.0 (7.0, 9.0) [*n* = 2244]
TMT‐A (Processing speed) [sec]	37.0 (30.0, 47.0) [*n* = 2185]
TMT‐B (Executive functioning) [sec]	81.0 (64.0, 105.0) [*n* = 2161]
MMSE sum score	28.0 (27.0, 29.0) [*n* = 2294]

Abbreviations: IQR, inter quartile range; MMSE, mini‐mental state examination; PSMD, Peak Width of Skeletonized Mean Diffusivity; TMT, trail‐making‐test; WMH, white matter hyperintensity.

### 3.2 Association of the BCS With Structural Brain Changes

After adjusting for age and sex, a 5‐point increase in BCS was associated with a lower WMH burden (relative increase 0.810; 95% CI [0.751, 0.874]) and a lower PSMD (relative increase 0.970; 95% CI [0.960, 0.980]), indicating reduced vascular lesion load and better structural integrity of the white matter. No associations were observed between BCS with cortical thickness and brain volume (Table 3; Figure 2).

**TABLE 3:** Associations between BCS and structural brain outcomes. Estimates represent the change per 5‐unit increase in BCS adjusted for age, sex, and their interaction, reported as absolute increases in the respective unit or as relative increases for log‐transformed outcomes.

Outcome	Estimated effect		95% confidence interval		*p*	*n*
			Lower bound	Upper bound		
Brain volume, normalized	Absolute increase of	0.002	−0.001	0.005	0.127	2356
WMH load [proportion of total brain volume ×10^3^]	Relative increase of	0.810	0.751	0.874	< 0.001	2318
Cortical thickness [mm]	Absolute increase of	0.007	0.000	0.014	0.061	2356
PSMD [×10^3^]	Relative increase of	0.970	0.960	0.980	< 0.001	2256
Global efficiency	Absolute increase of	0.004	0.001	0.006	0.004	2144
Global clustering coefficient	Relative increase of	0.978	0.969	0.988	< 0.001	2144

Abbreviations: PSMD, Peak Width of Skeletonized Mean Diffusivity; WMH, white matter hyperintensity.

**FIGURE 2:**
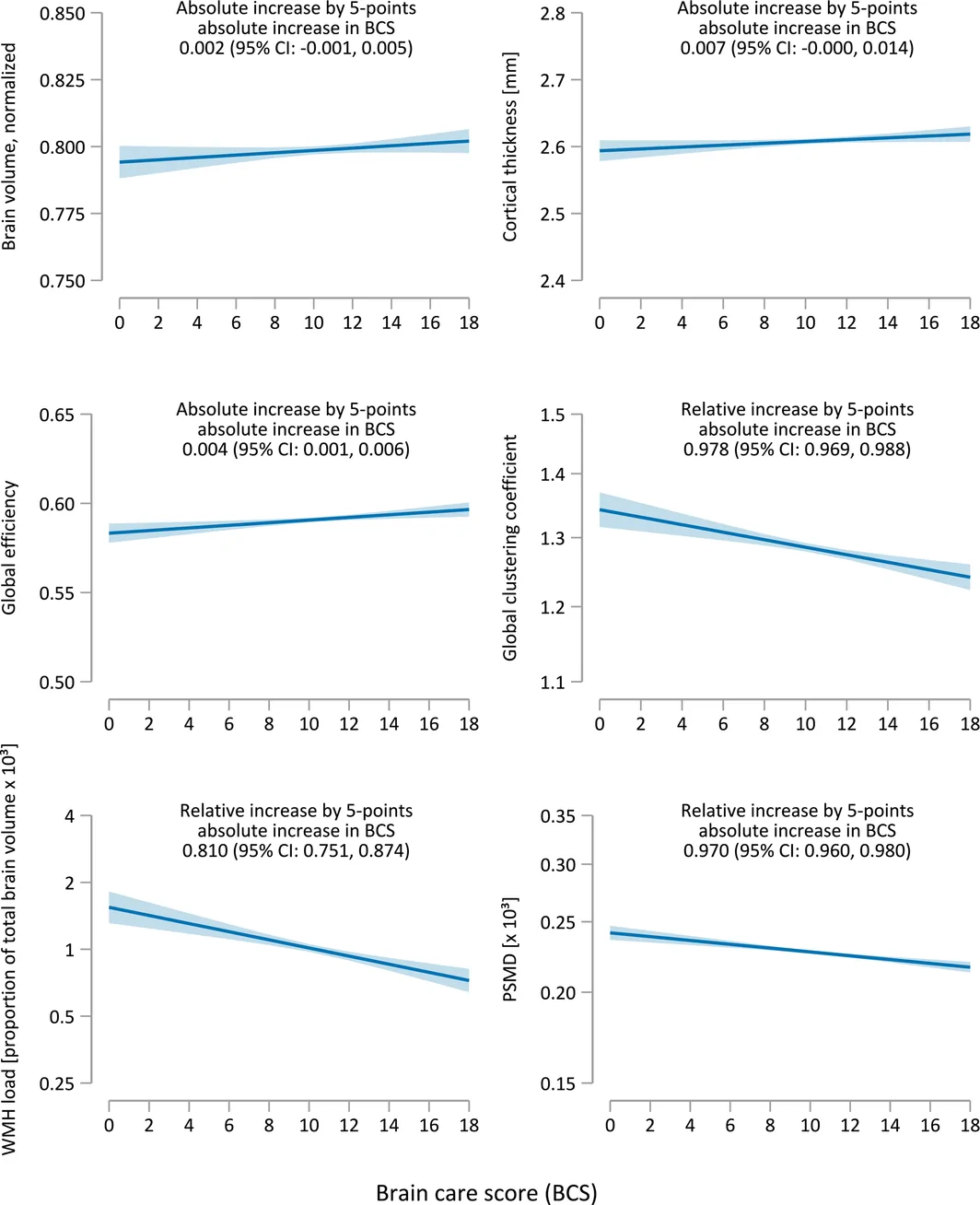
Estimated associations of the BCS with structural brain outcomes. Estimates and 95% confidence intervals represent the effect per 5‐unit increase in BCS, derived from linear regression models adjusted for age, sex, and their interaction. Results are reported as absolute increases in the respective unit or as relative increases for log‐transformed outcomes. PSMD, Peak Width of Skeletonized Mean Diffusivity; WMH, white matter hyperintensity.

### 3.3 Association of the BCS With Structural Brain Connectivity

A 5‐point increase in BCS was associated with a slight increase in global efficiency (absolute increase 0.004, 95% CI [0.001, 0.006]) and a decrease in the global clustering coefficient (relative increase 0.987, 95% CI [0.969, 0.988]) after adjusting for covariates (Table 3; Figure 2).

### 3.4 Association of the BCS With Cognitive Function

Cognitive performance across the cohort was generally preserved with a median MMSE score of 28 (IQR 27, 29). A 5‐point increase in BCS was associated with faster processing speed, as indicated by TMT‐A (relative increase 0.959, 95% CI [0.935, 0.983]), and with better executive function, reflected by TMT‐B completion times (relative increase 0.940, 95% CI [0.914, 0.967]). Similarly, a 5‐point increase in BCS was associated with higher verbal fluency, assessed by the Animal Naming Test (absolute increase 0.575, 95% CI [0.046, 1.104]) (Table 4; Figure 3).

**TABLE 4:** Associations between BCS and cognition test outcomes. Estimates represent the change per 5‐unit increase in BCS adjusted for age, sex, and their interaction, reported as absolute increases in the respective unit or as relative increases for log‐transformed outcomes.

Outcome	Estimated effect		95% confidence interval		*p*	*n*
			Lower bound	Upper bound		
Verbal fluency [*n* words]	Absolute increase of	0.575	0.046	1.104	0.033	2312
Processing speed [s]	Relative increase of	0.959	0.935	0.983	< 0.001	2185
Executive functioning [s]	Relative increase of	0.940	0.914	0.967	< 0.001	2161
Verbal memory [*n* words]	Absolute increase of	0.087	−0.052	0.226	0.218	2244
MMSE sum score	Absolute increase of	0.114	−0.018	0.245	0.091	2294

Abbreviation: MMSE, Mini‐Mental State Examination.

**FIGURE 3:**
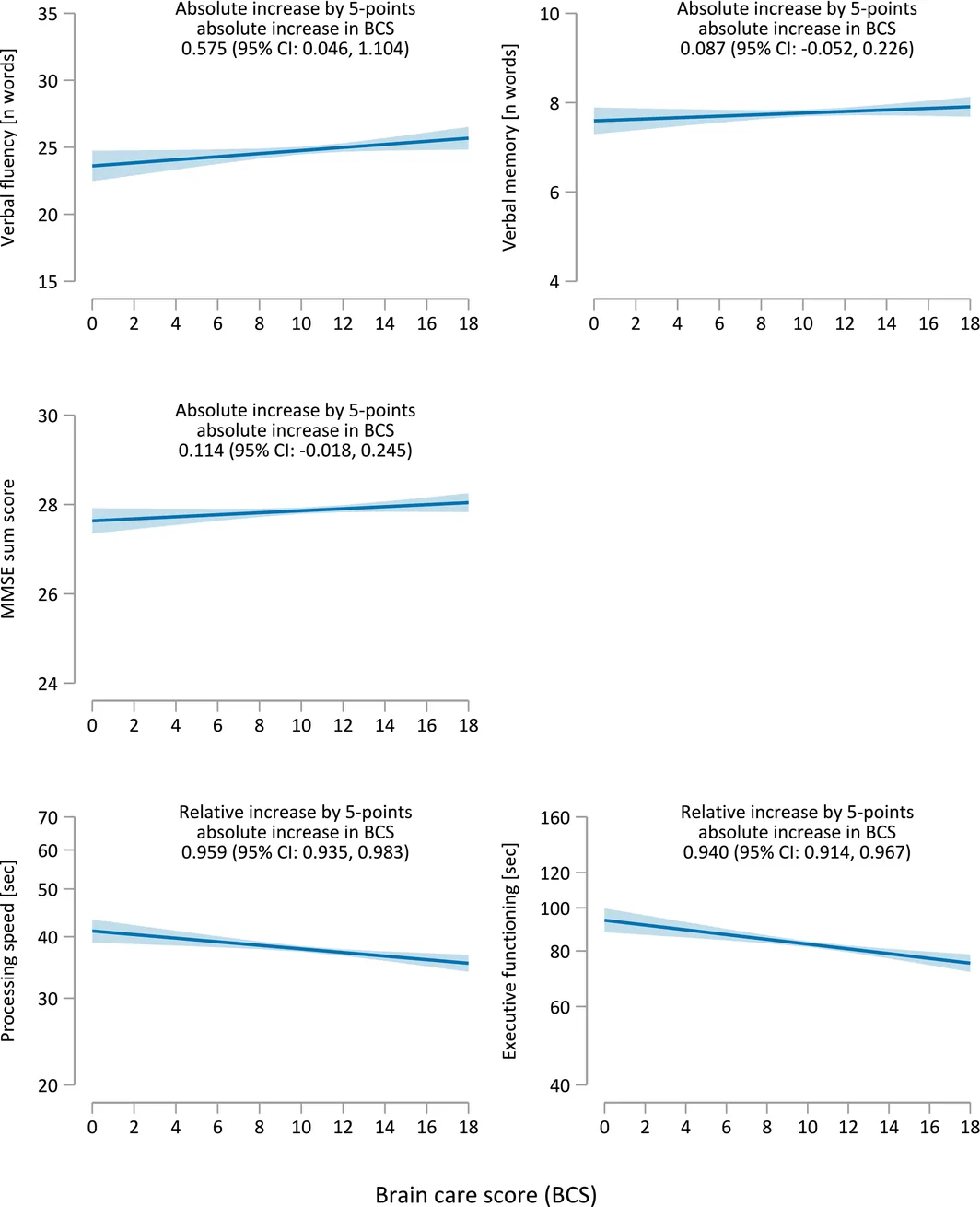
Estimated associations of the BCS with cognition test outcomes. Estimates and 95% confidence intervals represent the effect per 5‐unit increase in BCS, derived from linear regression models adjusted for age, sex, and their interaction. Results are reported as absolute increases in the respective unit or as relative increases for log‐transformed outcomes. MMSE, Mini‐Mental State Examination.

### 3.5 Sensitivity Analysis (Complete Case Analysis)

In the complete case analysis (*n* = 1971), associations between a 5‐point increase in BCS and markers of vascular brain damage were largely confirmed, including lower WMH burden (relative increase 0.798; 95% CI [0.731, 0.872]) and a lower PSMD (relative increase 0.967; 95% CI [0.955, 0.979]). Similarly, a 5‐point increase in BCS was associated with an increase in global efficiency (absolute increase 0.004, 95% CI [0.001, 0.007]) and a decrease in the global clustering coefficient (relative increase 0.976, 95% CI [0.965, 0.987]). No statistically significant associations were observed between the BCS and either neurodegenerative markers or cognitive performance in this analysis (Tables S1–S3). Details of the characteristics of the study population are shown in Table S1.

## 4 Discussion

In this cross‐sectional analysis of a population‐based sample of middle‐aged to elderly individuals, we confirmed the previously reported association between the BCS and structural brain changes and cognitive performance and extended existing evidence by demonstrating a novel association between the BCS and structural brain network organization. Our key findings are as follows: (1) a higher BCS was associated with less vascular brain damage, reflected by lower WMH burden and lower PSMD, whereas no associations were observed for brain volume and cortical thickness as markers of neurodegeneration; (2) a higher BCS was associated with a pattern of preserved structural connectivity, characterized by higher global efficiency and a lower clustering coefficient; and (3) a higher BCS was associated with better performance across multiple cognitive domains, including processing speed, executive function, and verbal fluency, although effect sizes were modest. There was no association of the BCS with verbal memory. A sensitivity analysis including only participants without any missing BCS items largely confirmed these findings with significant associations between the BCS and structural brain changes. There were no significant associations of the BCS with cognitive measures in this complete‐case analysis, although observed effects were in the same direction.

Our results align with previous findings from the UK Biobank, where individuals with higher BCS values showed lower WMH burden, higher fractional anisotropy, lower mean diffusivity, and slower progression of white matter abnormalities over time in a mid‐to‐late‐life, nationally recruited cohort [16]. We could confirm the cross‐sectional association of a higher BCS with lower WMH burden in the HCHS cohort, despite its smaller and regionally defined population. We extend these observations by a comprehensive analysis of the association of BCS with further imaging markers of vascular damage (PSMD), neurodegeneration (brain volume, cortical thickness), and brain network organization (global efficiency, global clustering coefficient).

In this analysis, a higher BCS was associated with lower PSMD, reflecting better white matter microstructural integrity. PSMD is an established marker of white matter microstructural damage with prognostic relevance in cognitive impairment and dementia, potentially mediating the relationship between cardiovascular risk and cognitive decline [32]. Markers of neurodegeneration, such as cortical thickness and brain volume, showed no associations with the BCS. This may reflect the predominantly vascular nature of the modifiable risk factors captured by the BCS, which we hypothesize act primarily through vascular brain damage rather than directly influencing neurodegenerative processes. Moreover, neurodegenerative changes may only become detectable at more advanced disease stages [33] than those represented in our sample, which predominantly consists of cognitively unimpaired individuals with limited variability in brain pathology. Structural MRI may lack the sensitivity to detect subtle early neurodegenerative changes, as macrostructural markers become abnormal only relatively late in the neurodegenerative disease process [34].

WMH are known to affect structural brain connectivity [6, 7, 35]. Using DTI‐based network analysis, we characterized structural brain network topology in terms of integration (global efficiency) and segregation (global clustering coefficient). As a novel finding regarding the association of the BCS with brain structure, we observed a modest increase in global efficiency alongside a reduction in the global clustering coefficient with higher BCS values. This pattern suggests a relative preservation of long‐range connections and less disruption of major white matter pathways, indicating a higher capacity for efficient transmission of information across distant brain regions in individuals with higher BCS. These findings are consistent with previous work demonstrating that increasing CSVD burden is associated with decreased integration and increased segregation of structural brain networks, reflecting a disruption of efficient network organization [6]. Similar alterations in network topology, characterized by reduced global efficiency and increased segregation, have also been reported in other conditions affecting structural brain connectivity, such as stroke and healthy aging [36, 37, 38, 39]. Furthermore, network‐based measures have been shown to predict cognitive performance more accurately than conventional lesion volumes alone, highlighting the importance of preserved network integrity for cognitive function [3]. Together with reduced vascular brain damage, preserved brain network integrity may represent a pathway linking higher BCS values to better cognitive function.

Previous studies have underscored the role of vascular risk factors in the development of CSVD and cognitive decline [40]. Although an association between BCS and cognitive impairment or dementia has been reported [12], prior work has not examined performance across specific cognitive domains. In our cohort, a higher BCS was consistently associated with better cognitive performance across multiple domains, including better verbal fluency, faster processing speed, and better executive functioning. These results suggest that the BCS captures aspects of vascular, metabolic, and lifestyle health that support cognitive function even in a relatively healthy population. However, the observed effect sizes were small and there was high heterogeneity in the endpoints observed in the data, which may reflect the generally healthy nature of this population‐based sample and the absence of overt cognitive impairment in most participants. We did not observe an association of the BCS with verbal memory. As impairment of verbal memory represents a hallmark and frequently early symptom of Alzheimer's dementia, this, on a behavioral level, reflects the lack of an association with imaging markers of neurodegeneration. Thus, our findings might point towards a specific sensitivity of the BCS to capture risk markers of vascular brain damage and resulting cognitive impairments, while it appears less sensitive to specific imaging and clinical findings of neurodegeneration. The associations with cognitive measures in the complete‐case analysis remained directionally consistent, but associations were not statistically significant, and the estimated effects were markedly attenuated relative to the primary analysis. This may result from reduced power resulting from smaller numbers, but we cannot rule out that our approach of missing‐data handling has also contributed to inflating associations with cognitive function.

The observed associations are biologically plausible and can be linked to specific components of the BCS. Hypertension impairs endothelial function and promotes white matter injury, thereby contributing to cerebrovascular and cognitive decline [41]. Diabetes exacerbates vascular endothelial dysfunction through mechanisms driven by oxidative stress [42]. These vascular risk factors are well‐established drivers of CSVD and are directly captured by the BCS, which may explain its association with imaging markers of vascular brain damage. Other pathogenetic pathways may additionally underlie the effects of modifiable risk factors included in the BCS.

Regular physical exercise enhances cerebral perfusion and mitigates microvascular damage, though the magnitude of these effects may depend on exercise intensity and cardiorespiratory fitness [43]. In addition, it has been shown that physical exercise can promote neuroplasticity via alterations in synaptic structure and function and support angiogenesis and glial activity, which may contribute to attenuated neurodegeneration and improvement of cognitive function [44]. Adherence to a balanced diet enriched with vegetables and fruits supports structural brain integrity, including preserved microstructural organization, increased cortical thickness and reduced WMH burden [45]. In a population‐based study of 1487 older adults, higher pro‐inflammatory dietary patterns were associated with higher risk of all‐cause dementia and AD, suggesting that anti‐inflammatory dietary patterns may provide neuroprotection through neuroinflammatory pathways [46]. Adequate sleep may further support brain health through the glymphatic system, which facilitates clearance of metabolic waste products during sleep, with chronic sleep disruption associated with increased susceptibility to neurodegenerative processes [47]. Psychosocial determinants, including stress management and social engagement, may further enhance resilience against cerebrovascular injury [48]. The multidimensional composition of the BCS allows these synergistic effects to be captured, providing a broader perspective than traditional vascular risk scores such as the Framingham Risk Score, the CAIDE Dementia Risk Score, or ASCVD‐based measures [13, 14].

Whether the BCS can be used for guiding individual behavior or preventive interventions remains to be established. Previous reports have already demonstrated the predictive value of the BCS for adverse outcomes including stroke, dementia, and depression [15, 17, 49]. Moreover, recent evidence suggests that higher BCS values may also be associated with a lower risk of cardiovascular disease and common cancers, indicating that the modifiable factors captured by BCS may reduce the risk of multiple chronic diseases beyond stroke and dementia [50]. Future studies should focus on validating the BCS in diverse and longitudinal cohorts to assess its predictive utility over time. Interventional trials targeting modifiable factors incorporated in the BCS are warranted to evaluate whether improvements translate into reduced CSVD progression, enhanced cognitive function, and lower incidence of stroke and dementia.

Some limitations of this study should be considered. As the categories “social relationships” and “meaning in life” were unavailable, a modified BCS was calculated. As a result, our analysis may underestimate the effects of the social emotional domain of the BCS. This also happened for the calculation of the BCS from UK Biobank data and should motivate a dedicated assessment of these measures in future population‐based studies.

The cross‐sectional design does not allow conclusions about mediation or causal relationships, and residual confounding cannot be entirely excluded. Moreover, self‐reported lifestyle factors such as diet, physical activity, and sleep may be subject to recall bias. Participants were recruited from a single urban region, which may limit generalizability to broader or more diverse populations. Furthermore, as a result of our strategy of recruiting participants, 32% of participants had a Framingham Stroke Score > 7, which may limit the generalizability of our findings to populations with lower vascular risk burden. Additionally, we were unable to assess changes in BCS over time, which would be necessary to evaluate whether improvements in the score translate into measurable changes in brain structure or cognitive function.

In conclusion, our findings demonstrate that the BCS is associated with imaging markers of structural brain health, brain network organization, and cognitive performance. The BCS captures modifiable physiological, lifestyle, and social risk factors that are linked to the pathophysiological mechanisms underlying cerebrovascular disease and dementia. By identifying individuals at elevated risk of CSVD, cognitive decline, and cerebrovascular events, the BCS may serve as a valuable instrument to guide behavioral change and support preventive strategies aimed at reducing the burden of stroke and dementia. Prospective longitudinal studies are needed to clarify potential causal relationships and to further investigate the temporal dynamics of the observed associations.

## Author Contributions

**Jenna Schellin:** writing – original draft, conceptualization, methodology, writing – review and editing, data curation. **Susanne Sehner:** formal analysis, methodology, writing – review and editing, visualization. **Marie Neumann:** investigation, writing – review and editing. **Marvin Petersen:** validation, writing – review and editing. **Felix Leonard Nägele:** validation, writing – review and editing. **Sanjula D. Singh:** writing – review and editing. **Jonathan Rosand:** writing – review and editing. **Nils Schumacher:** writing – review and editing. **Birgit‐Christiane Zyriax:** writing – review and editing. **Raphael Twerenbold:** writing – review and editing. **Bastian Cheng:** writing – review and editing, validation. **Götz Thomalla:** conceptualization, writing – review and editing, funding acquisition. **Märit Jensen:** supervision, writing – review and editing, conceptualization, methodology, data curation.

## Funding

The participating institutes and departments from the University Medical Center Hamburg‐Eppendorf contribute all with individual and scaled budgets to the overall funding of the Hamburg City Health Study.

## Conflicts of Interest

M.J. reports personal fees from Bristol‐Myers Squibb and Bayer. M.J. is a fellow of the Hertie Network of Excellence in Clinical Neuroscience. G.T. reports personal fees as consultant or lecturer from Acandis, Astra Zeneca, Bayer, Boehringer Ingelheim, Bristol Myers Squibb/Pfizer, Daiichi Sankyo, Lilly, and Stryker. All other authors report no conflicts of interest.

## Supporting information


**Table S1:** Comparison of the study population of the primary analysis and the complete case analysis.
**Table S2:** Sensitivity analysis with a complete‐case population for all BCS items: Associations between BCS and structural brain outcomes. Estimates represent the change per 5‐unit increase in BCS adjusted for age, sex, and their interaction, reported as absolute increases in the respective unit or as relative increases for log‐transformed outcomes.
**Table S3:** Sensitivity analysis with a complete‐case population for all BCS items: Associations between BCS and cognition test outcomes. Estimates represent the change per 5‐unit increase in BCS adjusted for age, sex, and their interaction, reported as absolute increases in the respective unit or as relative increases for log‐transformed outcomes.
**Table S4:** Missing pattern (physical subscale) for patients with available MRI (*N* = 2618).
**Table S5:** Missing pattern (lifestyle subscale) for patients with available MRI (*N* = 2618).
**Table S6:** Missing pattern (social/emotional subscale) for patients with available MRI (*N* = 2618).
**Figure S1:** Scatterplot between BCS and structural brain outcomes. Scatterplots show the raw data points alongside the estimated linear regression fit (blue line), adjusted for age, sex, and their interaction. The orange line represents the local polynomial regression fit (local mean smoothing, Epanechnikov kernel, bandwidth = 0.8) used to visually assess linearity. Shaded areas indicate the 95% confidence intervals for both models.
**Figure S2:** Scatterplot between BCS and cognition test outcomes. Scatterplots show the raw data points alongside the estimated linear regression fit (blue line), adjusted for age, sex, and their interaction. The orange line represents the local polynomial regression fit (local mean smoothing, Epanechnikov kernel, bandwidth = 0.8) used to visually assess linearity. Shaded areas indicate the 95% confidence intervals for both models. MMSE, Mini‐Mental State Examination.

## Data Availability

HCHS data can be obtained by qualified researchers on reasonable request to the study's steering committee.
