# Supplementary material for: Associations Between the Brain Care Score and Structural Brain Changes and Cognitive Impairment Using Data From the Hamburg City Health Study
Source: Eur J Neurol. 2026 Oct 22;33(10):e70796. doi: 10.1111/ene.70796 (PMC13691427; doi:10.1111/ene.70796)
Supplement: Supplementary file 1 [file ENE-33-e70796-s001.docx]

**Supplementary material**

**Associations between the Brain Care Score and Structural Brain Changes and Cognitive Impairment Using Data from the Hamburg City Health Study**

Jenna Schellin¹, Susanne Sehner², Marie Neumann², Marvin Petersen¹, Felix Leonard Nägele¹, Sanjula D. Singh⁴˒⁵, Jonathan Rosand⁴˒⁵, Nils Schumacher⁶, Birgit-Christiane Zyriax³, Raphael Twerenbold², Bastian Cheng¹, Götz Thomalla¹, Märit Jensen¹

**Affiliations:**
¹ Department of Neurology, University Medical Center Hamburg-Eppendorf (UKE), Hamburg, Germany
² Department of Cardiology, University Medical Center Hamburg-Eppendorf (UKE), Hamburg, Germany
³ Research Group Preventive Medicine and Nutrition, Midwifery Science, Health Care Research and Prevention, University Medical Center Hamburg-Eppendorf (UKE), Hamburg, Germany
⁴ Brain Care Labs, Department of Neurology, Massachusetts General Hospital, Harvard Medical School, Boston, MA, USA
⁵ Broad Institute of MIT and Harvard, Cambridge, MA, USA
⁶ Institute of Human Movement Science, Science and Transfer Center Active City, University of Hamburg, Hamburg, Germany

**Table of contents**

| Supplementary Table S1: Comparison of the study population of the primary analysis and the complete case analysis | p. 2-3 |
| --- | --- |
| Supplementary Table S2: Sensitivity analysis with a complete-case population for all BCS items: Associations between BCS and structural brain outcomes. | p. 4 |
| Supplementary Table S3: Sensitivity analysis with a complete-case population for all BCS items: Associations between BCS and cognition test outcomes | p. 4 |
| Supplementary Tables S4-S6: Missing patterns | p. 5 |
| Supplementary Figure S1: Scatterplot between BCS and structural brain outcomes | p. 6 |
| Supplementary Figure S2: Scatterplot between BCS and cognition test outcomes | p. 7 |

**Supplementary tables**

**Table S1:** Comparison of the study population of the primary analysis and the complete case analysis

| **Characteristic** | Primary analysis (N =2,453) | Complete case analysis (N =1,971) |
| --- | --- | --- |
| Age [years], mean (SD) | 63.8 (8.4) | 63.8 (8.4) |
| Female, n/N (%) | 1,075/2,453 (43.8%) | 830/1,971 (42.1%) |
| Level of education, n/N (%) |  |  |
| low education | 96/2,369 (4.1%) | 67/1,916 (3.5%) |
| medium education | 1,158/2,369 (48.9%) | 910/1,916 (47.5%) |
| high education | 1,115/2,369 (47.1%) | 939/1,916 (49.0%) |
| **Medical history and risk factors**, n/N (%) |  |  |
| Arterial hypertension | 1,711/2,372 (72.1%) | 1,375/1,933 (71.1%) |
| Diabetes mellitus | 210/2,296 (9.1%) | 168/1,890 (8.9%) |
| Hypercholesterolaemia | 619/2,311 (26.8%) | 487/1,897 (25.7%) |
| Atrial fibrillation | 136/2,250 (6.0%) | 114/1,851 (6.2%) |
| Metabolic syndrome | 958/2,174 (44.1%) | 792/1,810 (43.8%) |
| Smoking |  |  |
| never/past | 2,024/2,446 (82.7%) | 1,637/1,971 (83.1%) |
| current | 422/2,446 (17.3%) | 334/1,971 (16.9%) |
| History of myocardial infarction | 85/2,443 (3.5%) | 69/1,968 (3.5%) |
| History of heart failure | 110/2,268 (4.9%) | 95/1,844 (5.2%) |
| History of stroke | 75/2,439 (3.1%) | 55/1,964 (2.8%) |
| **Brain Care Score (0-18)**, median **(**IQR) | 11.0 (9.0,12.0) | 11.0 (9.0,13.0) |
| Category Physical (potential range 0-8) | 4.0 (3.0,5.0) | 4.0 (3.0,5.0) |
| Category Lifestyle (potential range 0-9) | 6.0 (4.0,7.0) | 6.0 (5.0,7.0) |
| Category Social/Emotional (potential range 0-1), n (%) | 1 Point: 1,963/2,453 (80.0%)  0 Points: 490/2,453 (20.0%) | 1 Point: 1,590/1,971 (80.7%)  0 Points: 381/1,971 (19.3%) |
| **Brain Imaging parameters**, median (IQR) |  |  |
| Brain volume, normalized | 0.80 (0.77, 0.83) [n=2,356] | 0.80 (0.77-0.83) [n=1,894] |
| WMH load [proportion of total brain volume x 10^3^] | 0.99 (0.51, 1.98) [n=2,318] | 0.97 (0.50-1.95) [n=1,869] |
| PSMD [x 10^3^] | 0.22 (0.20, 0.25) [n=2,256] | 0.22 (0.20-0.25) [n=1,806] |
| Cortical thickness [mm] | 2.61 (2.55, 2.67) [n=2,356] | 2.61 (2.55-2.68) [n=1,894] |
| Global efficiency | 0.59 (0.57, 0.61) [n=2,144] | 0.59 (0.57-0.61) [n=1,723] |
| Global clustering coefficient | 1.24 (1.20, 1.31) [n=2,144] | 1.24 (1.20-1.31) [n=1,723] |
| **Cognitive assessment**, median (IQR) |  |  |
| Animal naming test (Verbal fluency)  [n words] | 24.0 (20.0, 29.0) [n=2,312] | 25.0 (20.0-29.0) [n=1,862] |
| Word list recall (Verbal memory) [n words] | 8.0 (7.0, 9.0) [n=2,244] | 8.0 (7.0-9.0) [n=1,810] |
| TMT-A (Processing speed) [sec] | 37.0 (30.0, 47.0) [n=2,185] | 37.0 (30.0-46.0) [n=1,776] |
| TMT-B (Executive functioning) [sec] | 81.0 (64.0, 105.0) [n=2,161] | 80.0 (63.0-103.0) [n=1,758] |
| MMSE sum score | 28.0 (27.0, 29.0) [n=2,294] | 28.0 (27.0-29.0) [n=1,849] |

Abbreviations: IQR, Inter Quartile Range; WMH, White Matter Hyperintensity; PSMD, Peak Width of Skeletonized Mean Diffusivity; TMT, Trail-Making-Test; MMSE, Mini–Mental State Examination.

**Table S2:** Sensitivity analysis with a complete-case population for all BCS items: Associations between BCS and structural brain outcomes. Estimates represent the change per 5-unit increase in BCS adjusted for age, sex, and their interaction, reported as absolute increases in the respective unit or as relative increases for log-transformed outcomes.

| **Outcome** | **Estimated effect** | | **95% confidence interval** | | **p-value** | **n** |
| --- | --- | --- | --- | --- | --- | --- |
|  |  |  | **lower bound** | **upper bound** |  |  |
| **Brain volume, normalized** | absolute increase of | 0.001 | -0.002 | 0.005 | 0.416 | 1,894 |
| **WMH load [proportion of total brain volume x 10^3^]** | relative increase of | 0.810 | 0.798 | 0.872 | <0.001 | 1,869 |
| **Cortical thickness [mm]** | absolute increase of | 0.007 | -0.002 | 0.015 | 0.107 | 1,894 |
| **PSMD [x 10^3^]** | relative increase of | 0.967 | 0.955 | 0.979 | <0.001 | 1,806 |
| **Global efficiency** | absolute increase of | 0.004 | 0.001 | 0.007 | 0.009 | 1,723 |
| **Global clustering coefficient** | relative increase of | 0.976 | 0.965 | 0.987 | <0.001 | 1,723 |

Abbreviations: WMH, White Matter Hyperintensity; PSMD, Peak Width of Skeletonized Mean Diffusivity.

**Table S3:** Sensitivity analysis with a complete-case population for all BCS items: Associations between BCS and cognition test outcomes. Estimates represent the change per 5-unit increase in BCS adjusted for age, sex, and their interaction, reported as absolute increases in the respective unit or as relative increases for log-transformed outcomes.

| **Outcome** | **Estimated effect** | | **95% confidence interval** | | **p-value** | **n** |
| --- | --- | --- | --- | --- | --- | --- |
|  |  |  | **lower bound** | **upper bound** |  |  |
| **Verbal fluency [n words]** | absolute increase of | 0.272 | -0.347 | 0.892 | 0.389 | 1,862 |
| **Processing speed [sec]** | relative increase of | 0.980 | 0.952 | 1.009 | 0.176 | 1,776 |
| **Executive functioning [sec]** | relative increase of | 0.972 | 0.940 | 1.004 | 0.083 | 1,758 |
| **Verbal memory [n words]** | absolute increase of | 0.102 | -0.060 | 0.264 | 0.218 | 1,810 |
| **MMSE sum score** | absolute increase of | 0.126 | -0.026 | 0.279 | 0.104 | 1,849 |

Abbreviations: MMSE, Mini-Mental State Examination.

### **Table S4:** Missing pattern (physical subscale) for patients with available MRI (N=2,618)

| **N** | **Percent** | **Systolic blood pressure** | **Diastolic blood pressure** | **HbA1c (Blood glucose)** | **Cholesterol** | | **Body Mass Index** |
| --- | --- | --- | --- | --- | --- | --- | --- |
| 2383 | 91.02 |  |  |  | |  |  |
| 82 | 3.13 |  |  |  | |  | mis |
| 69 | 2.64 | mis | mis |  | |  |  |
| 47 | 1.80 |  |  | mis | | mis |  |
| 21 | 0.80 |  |  | mis | |  |  |
| 7 | 0.27 |  |  |  | | mis |  |
| 4 | 0.15 |  |  | mis | | mis | mis |
| 3 | 0.11 | mis | mis |  | |  | mis |
| 1 | 0.04 | mis | mis | mis | | mis |  |
| 1 | 0.04 | mis | mis |  | | mis |  |

### **Table S5:** Missing pattern (lifestyle subscale) for patients with available MRI (N=2,618)

| **N** | **Percent** | **Nutrition** | **Alcohol consumption** | **Smoking** | **Aerobic activities** | **Sleep** |
| --- | --- | --- | --- | --- | --- | --- |
| 2214 | 84.57 |  |  |  |  |  |
| 129 | 4.93 | mis | mis |  | mis | mis |
| 100 | 3.82 |  | mis |  |  |  |
| 53 | 2.02 |  |  |  | mis | mis |
| 27 | 1.03 |  |  |  | mis |  |
| 26 | 0.99 |  |  |  |  | mis |
| 24 | 0.92 |  | mis |  | mis | mis |
| 19 | 0.73 | mis | mis |  |  |  |
| 12 | 0.46 |  | mis |  | mis |  |
| 7 | 0.27 |  |  | mis |  |  |
| 4 | 0.15 |  | mis |  |  | mis |
| 2 | 0.08 | mis | mis |  | mis |  |
| 1 | 0.04 | mis | mis | mis | mis | mis |

### **Table S6:** Missing pattern (social/emotional subscale) for patients with available MRI (N=2,618)

| **N** | **Percent** | **Stress** |
| --- | --- | --- |
| 1499 | 57.26 |  |
| 954 | 36.44 |  |
| 165 | 6.3 | mis |

**Figure S1: Scatterplot between BCS and structural brain outcomes.** Scatterplots show the raw data points alongside the estimated linear regression fit (blue line), adjusted for age, sex, and their interaction. The orange line represents the local polynomial regression fit (local mean smoothing, Epanechnikov kernel, bandwidth = 0.8) used to visually assess linearity. Shaded areas indicate the 95% confidence intervals for both models.

Abbreviations: WMH, White Matter Hyperintensity; PSMD, Peak Width of Skeletonized Mean Diffusivity.

**Figure S2: Scatterplot between BCS and cognition test outcomes.** Scatterplots show the raw data points alongside the estimated linear regression fit (blue line), adjusted for age, sex, and their interaction. The orange line represents the local polynomial regression fit (local mean smoothing, Epanechnikov kernel, bandwidth = 0.8) used to visually assess linearity. Shaded areas indicate the 95% confidence intervals for both models.
Abbreviations: MMSE, Mini-Mental State Examination.
